# France and America

**Published:** 1879-02

**Authors:** 


### FRANCE IN AMERICA.

"SILENCE à l'orgie"—freely Anglicized, "Shut your mouth,"—was the famous cry of a Frenchman, against a certain class of outlawed literateurs, "for their revelry in obscenities, and their clamoring after personalities;" and types of these we find in our own country, both in the secular and the religious press, and to an extent which ought to arrest the attention of all conservative men. The apostrophe of the beautiful and gifted Madam Roland, as she bared her neck to the guillotine, finds an echo in Christian America: "O, Liberty! how many crimes are perpetrated in thy name!" Even in America, the liberty of the press is daily becoming more and more a license and a nuisance. Even among good men, useful men, honored men, there is an impatience of opposition, in mere difference of opinion, which is astounding. A man can scarcely utter his sentiments on any debatable subject, but, in three cases out of four, he will be assailed with epithets, with personalities, with an impugnment of motives, which leaves us in doubt whether the assailants are from the gutter or from bedlam. Instead of a quiet, calm, dignified, and confident marching-up argument, there is the primary befooling, the contemptible quibble, the silly jest, the malignant sarcasm, and then the mean personalities, with a persistent effort, all through, to be tremendously severe.

In any newspaper controversy, civil or religious, nine men out of ten will not fail to show, within three passes, that their origin was the mud-puddle, not three generations a-gone, not two, often direct. As for the man who can meet weapons like these with calm indifference, with silent contempt, and will steadily throw in his fire without noise, or trickery, or roundabouts—where is he, among a million?

Americans brag a great deal about their bravery. Brave people never brag. True courage is always quiet. Bluster and bravado are the native elements of the born poltroon. True courage wants nothing more than a clear field and a fair fight. There are few editors who have not witnessed, with an amusing contempt, the willingness of correspondents to say hard things in the dark, standing behind them, as cowardly Indians shoot from behind trees. There is as much degradation in aiming epithets and accusations at known personages from behind an anonyme, as there is in shooting through a window at night. Some of our great men can do these things; having been allowed to do so, no doubt, that, by the contemplation of what mean things they can do sometimes, they may never be puffed up, in their own estimation. In illustration, a fact of very recent occurrence may be given, which fully sets forth the truth of what has been said. One of the most eminent theologians of the age, and said to be as amiable as he is eminent, closes a nameless article, directed against a proposition made by another, his equal in many things, his superior in more, and who had been charged with the motive of desiring to have the agency in question, —it "is a task which none but an idiot or an angel would dare to undertake." To make such a shot at a man, known and good and honored everywhere, from the darkness, is a degradation to any one, whatever may be his greatness or his goodness.

"That is a diversion; let us go on with the argument," said Henderson, the Scotchman, after coolly wiping the water from his face, which his opponent, in his rage of inevitable defeat, had thrown from his glass. And quite as self-debased must the theologian have felt, as did Henderson's antagonist, when a reply came with all the dignity of a scholar, the forbearance of a Christian, and the high-bred courtesy of a gentleman born.

If, then, a Frenchman can feel outraged at the indecencies and degrading personalities of the press in his own country, it is high time that, in view of similar things here, we should take up the refrain, "Silence à l'orgie."

